# Epidemiological Principles in Claims of Causality: An Enquiry into Repetitive Head Impacts (RHI) and Chronic Traumatic Encephalopathy (CTE)

**DOI:** 10.1007/s40279-024-02102-4

**Published:** 2024-09-15

**Authors:** Lauren V. Fortington, J. David Cassidy, Rudolph J. Castellani, Andrew J. Gardner, Andrew S. McIntosh, Michael Austen, Zachary Yukio Kerr, Kenneth L. Quarrie

**Affiliations:** 1 School of Medical and Health Sciences, Edith Cowan University, Joondalup, WA Australia; 2 Division of Epidemiology, Dalla Lana School of Public Health, University of Toronto, Toronto, ON Canada; 3 Division of Neuropathology, Northwestern University Feinberg School of Medicine and Mesulam Center for Cognitive Neurology and Alzheimer’s Disease, Chicago, IL USA; 4 Sydney School of Health Sciences, Faculty of Medicine and Health, The University of Sydney, Camperdown, NSW Australia; 5 Monash University Accident Research Centre, Monash University, Clayton, VIC Australia; 6 Australasian Faculty of Occupational and Environmental Medicine, Royal Australasian College of Physicians, Sydney, Australia; 7 Royal New Zealand College of Urgent Care, Auckland, New Zealand; 8 High Court of New Zealand, Auckland, New Zealand; 9 Department of Exercise and Sport Science, University of North Carolina at Chapel Hill, Chapel Hill, NC USA; 10 New Zealand Rugby, 100 Molesworth Street, Wellington, New Zealand; 11 Sports Performance Research Institute New Zealand (SPRINZ), Auckland University of Technology, Auckland, New Zealand; 12 Auckland Bioengineering Institute (ABI), The University of Auckland, Auckland, Auckland, New Zealand

## Abstract

Determining whether repetitive head impacts (RHI) cause the development of chronic traumatic encephalopathy (CTE)-neuropathological change (NC) and whether pathological changes cause clinical syndromes are topics of considerable interest to the global sports medicine community. In 2022, an article was published that used the Bradford Hill criteria to evaluate the claim that RHI cause CTE. The publication garnered international media attention and has since been promoted as definitive proof that causality has been established. Our counterpoint presents an appraisal of the published article in terms of the claims made and the scientific literature used in developing those claims. We conclude that the evidence provided does not justify the causal claims. We discuss how causes are conceptualised in modern epidemiology and highlight shortcomings in the current definitions and measurement of exposures (RHI) and outcomes (CTE). We address the Bradford Hill arguments that are used as evidence in the original review and conclude that assertions of causality having been established are premature. Members of the scientific community must be cautious of making causal claims until the proposed exposures and outcomes are well defined and consistently measured, and findings from appropriately designed studies have been published. Evaluating and reflecting on the quality of research is a crucial step in providing accurate evidence-based information to the public.

**Graphical abstract:**

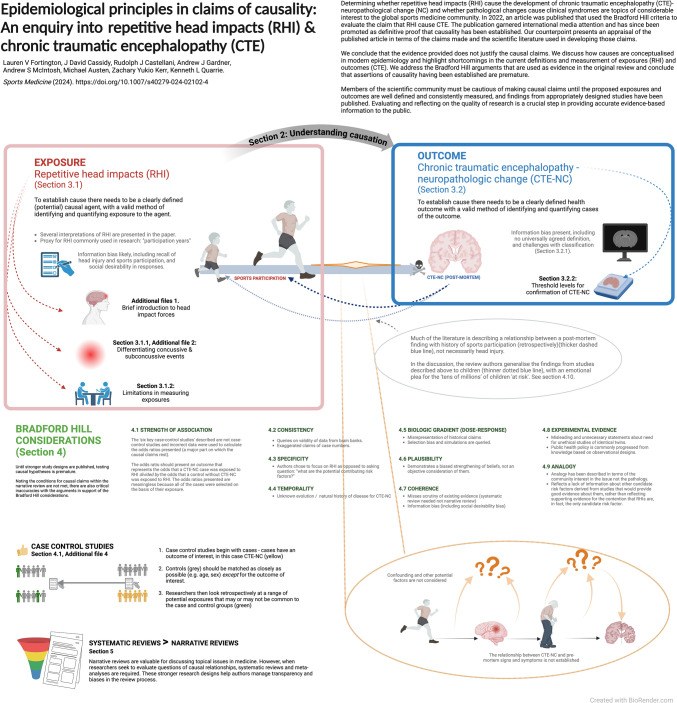

**Supplementary Information:**

The online version contains supplementary material available at 10.1007/s40279-024-02102-4.

## Key Points

**Table:** 

Exploring the causal links between repetitive head impacts (RHI) and chronic traumatic encephalopathy (CTE) is a topic of considerable interest globally. In July 2022, a paper claimed definitive evidence that RHI cause CTE.
It is part of the scientific process to examine all claims made by researchers and issue advocates and our counterpoint was designed to do this. We conclude that the evidence presented in the paper does not justify causal claims.
Members of the scientific community must be cautious of making or accepting causal claims until the proposed causes and health outcomes are well-defined, properly designed epidemiological studies have been undertaken, and the relationships between exposure to the proposed causes and the outcome(s) in which they are hypothesised to result have been accurately quantified.

## Introduction

In July 2022, a paper published in *Frontiers in Neurology* [1] claimed ‘conclusive evidence’ [2] that repetitive head impacts (RHI) were a ‘definitive cause’ [3] of chronic traumatic encephalopathy (CTE). Exploring the causal links between RHI and CTE is a topic of considerable interest globally, and unsurprisingly there was substantial media attention given to the publication. In their paper, the authors had applied the nine Bradford Hill criteria to the evidence they curated from the scientific literature to support their claim. They declared that certain microscopic phenomena [referred to hereafter as chronic traumatic encephalopathy neuropathologic change (CTE-NC)] found within autopsied brains could be directly attributed to exposure to RHI sustained earlier in life. In the conclusions section of their article, having made the caveat that all evidence is forever imperfect, they stated ‘After reviewing the medical literature on RHI and CTE through the Bradford Hill criteria, we have the highest confidence in the conclusion that RHI *causes* CTE’ [1, p 14].

It is part of the scientific process to critically examine all claims made by researchers and advocates; evaluating whether assertions are justified by the facts forms the basis of the peer-review system. It is with this spirit of critical enquiry that we have evaluated the causal claims made in the narrative review [1]. We aim to provide a counterpoint, through additional scrutiny of the supporting literature that was presented, and to consider how the authors’ claims hold against modern causal thinking in epidemiology.

This counterpoint is important because the assertion made in the original manuscript [1] has been accepted as fact in subsequent publications [4, 5] and in several influential settings, including the National Institute of Health in the USA [6] and an Australian Senate (national parliamentary) inquiry [7]. It is crucial that researchers strive to present objective, evidence-based information not only for the individuals who have experienced RHI, and/or fear the potential consequences of exposure to them [8], but also for those who rely on such evidence to inform policies and regulations, including practitioners in clinical medicine and/or public health, sports administrators, insurance actuaries, educators, lawmakers and the judiciary. To help readers navigate the concepts of causality presented in this manuscript, a visual overview is presented in the Graphical Abstract, with section numbers corresponding to the relevant text.

## Understanding Causation

*If, say, more than one factor is responsible for some effect, it is important that we do not pre-empt the scientific judgement: there is always the danger that we might refuse to admit any other ideas than the ones we happen to have at hand…* Sir Karl Popper [9].

The term ‘cause’ (along with ‘causal’, ‘causation’ and ‘causality’) has different interpretations in different professions. As an example, when considering a ‘cause of death’, the acceptance of evidence as causal differs in a court of law [10] to that required for completion of a death certificate [11]. In a topical scientific issue requiring consideration and investigation by biomechanists, psychologists, neuropathologists, sports medicine physicians, epidemiologists, sociologists and others, establishing and reaching consensus upon terms is a fundamental requirement.

Within epidemiology, the causal agent of injury is generally accepted to be ‘energy transfer’ [12]. For certain sports injuries, the cause and outcome can be obvious when there is an acute onset with instantaneous tissue failure, such as with a fractured lower leg, which results in immediate pain, functional impairment and visible bone damage. However, other injuries result not from a single identifiable event but from exposure to repetitive loads, and are considered to be ‘gradual onset’ in nature [13], (e.g. conditions such as ‘jumper’s knee’ or a bone stress injury).

Multiple contributory factors can play a role in the aetiology of both acute and gradual onset injuries and contribute to undesirable long-term outcomes. These factors include biomechanical load, age, genetics and environmental conditions, among others. A major goal of analytical epidemiology is to understand which factors are, and are not, on the causal pathway for a given health outcome. [14].

The complexity of the causal proposition at hand, that RHI causes a neurodegenerative disease, should not be underestimated. Notably, in the narrative review, the authors refer to CTE as a ‘neurodegenerative disease’, and thus the issue of an environmental cause (RHI) for a neurodegenerative disease is raised. Considerable uncertainty among experts remains about possible environmental causes of canonical neurodegenerative diseases (e.g. Alzheimer’s disease, Parkinson’s disease, frontotemporal dementia, amyotrophic lateral sclerosis) and in fact the causes of most neurodegenerative diseases are yet to be established despite significant research efforts [15, 16]. Dementia, which is an umbrella term describing the progressive cognitive impairments that accompany many neurodegenerative diseases, has been linked with a wide range of possible causes [17], and 12 risk factors identified through systematic reviews and meta-analyses have been suggested to account for approximately 40% of dementia cases world-wide [17]. The factors are ‘less education, hypertension, hearing impairment, smoking, obesity, depression, physical inactivity, diabetes, infrequent social contact, excessive alcohol consumption, head injury, and air pollution’ [17]. Risk factors are not necessarily ‘causes’—for example, hearing loss may be a cause, an early symptom or both of dementia [18, 19]. A small subset of neurodegenerative diseases are strongly hereditary, and known to be driven by genetic mutations (e.g. Huntington’s disease).

*Does exposure to A, either in isolation or in concert with other agents, cause B?*

Although this simple question captures the essence of what we want to know regarding causal relationships in epidemiology, philosophers of science from Hume [20] onwards have pointed out that drawing inferences from the specific to the general rests on inductive reasoning, and can thus only ever provide probabilistic evidence (in contrast to the logical certainty inherent in deductive reasoning based on Aristotelean syllogisms). Even so, causal pragmatists [21] following the ideas of Mill [22] hold that evidence of causation can be sufficiently well established to allow a basis for action via careful application of scientific methods to knowledge acquisition founded on systematic observation and experimentation.

In 1965, Bradford Hill set forth nine ‘viewpoints’ for evaluating whether evidence from associations via observational studies could be construed to be causal. These viewpoints were an expansion of causal criteria from a landmark report published by the U.S. Surgeon General on smoking and health. That report documented the results of 29 case–control and cohort studies from the UK and the USA that showed a very strong relationship between smoking and lung cancer (i.e. risk estimates over ten) [23]. Since that time there have been considerable further developments in epidemiology with respect to appraising whether causal claims are well supported by the evidence [24].

Modern epidemiology is based on testing competing theories (e.g. hypothesis testing) by conjecture (i.e. stating a hypothesis) and refutation (i.e. testing the hypothesis). Case reports, case series and cross-sectional study designs have an important role in generating hypotheses to be tested in more rigorous designs. For testing causal hypotheses, injury epidemiology relies mostly on observational designs such as case–control and cohort studies. [In some cases, causation can be inferred by testing injury prevention strategies in a randomised controlled trial (RCT). If mitigation of a risk factor in a RCT results in control or prevention of an outcome, it follows that the risk factor plays some role in the causal chain of events.]

The publication of the biopsychosocial model of the determinants of disease by Engel [25], and its subsequent evolution alongside contemporaneous work in the ‘new public health’ [26] and modern epidemiology by Rothman [27] (amongst others), highlighted that in real-world settings, multi-causality in the development of health and disease outcomes is the norm, rather than the exception. Recognition that complex interactions among multiple factors that may vary over time [28] was a feature of the aetiology of many diseases and health conditions (e.g. cancers, cardiovascular diseases and Alzheimer’s disease), resulted in new thinking about how to consider causation and drove developments in analytical methods in epidemiology that were able to deal with multiple time-dependent contributing and confounding factors [29, 30]. With this evolution, causal questions have extended beyond ‘does exposure to agent A cause outcome B?’ to ‘what conditions hold under which the causal relationship exists in the specified population/setting in the first place?’ The answer to multi-causal questions may help guide the best point of intervention for effective preventative efforts.

In practice, acceptance of a causal relationship (as opposed to the existence of a causal relationship) is a social phenomenon, resting upon the accrual, systematic synthesis and evaluation of factual evidence (and the absence of counter-evidence) from appropriately designed and conducted studies sufficient to satisfy subject-matter experts [31] in the scientific community [32] and society at large that the relationship under consideration is causal [33].

There are no criteria available against which epidemiological evidence can be set that allows researchers to state unequivocally that exposure to agent A causes outcome B. Bradford Hill explicitly recognised this fact:

*What I do not believe—and this has been suggested—is that we can usefully lay down some hard-and-fast rules of evidence that must be obeyed before we accept cause and effect. None of my nine viewpoints can bring indisputable evidence for or against the cause-and-effect hypothesis and none can be required as a sine qua non. What they can do, with greater or less strength, is to help us to make up our minds on the fundamental question—is there any other way of explaining the set of facts before us, is there any other answer equally, or more, likely than cause and effect?* [34]

Several theories and models for evaluating causal relationships in epidemiology exist, with most textbooks providing detailed descriptions of the required concepts [35 chapter 5, and other sections, 36 chapter 2, among other sections, 37 chapter 3, among other sections], summarised as:

- a clearly defined causal agent with a valid method of identifying/quantifying exposure to the agent;

that precedes

- a clearly defined health outcome with a valid method of identifying/quantifying cases of the outcome.

The causal agent and health outcome can then be considered through appropriate research designs that are capable of testing causal hypotheses in an unbiased manner.

Absence of these conditions means that the relationship between the potential causal agent and the outcome cannot be accurately determined and renders further consideration of a given causal claim otiose. As noted by Nieuwenhuijsen [38, p 5] in their text on exposure assessment in environmental epidemiology, ‘quantification of the relation between exposure and adverse human health effects requires the use of exposure estimates that are accurate, precise, and biologically relevant for the critical exposure period, and show a range of exposure levels in the population under study…’ It is for these reasons that epidemiologists take great care to create accurate definitions of the proposed agents and outcomes and consider how they will be measured as well as what hypothesis-testing research designs are best suited to study causal relationships.

## Gaps in the Presented Evidence

### Repetitive Head Impacts are not a Clearly Defined or Reliably Measured Causal Agent

In the review [1], the agent postulated to be the cause of CTE-NC is ‘repetitive head impacts’, defined as ‘the cumulative exposure to recurrent concussive and subconcussive events’ [1]. Please refer to Additional file 1: Introduction to head impact forces for background to this section.

#### Differentiating Concussive and Subconcussive Events

It is unclear exactly what the authors of the review [1] mean by ‘subconcussive’ or ‘concussive’ events, and whether the ‘event’ should be considered in relation to injury or independently. If ‘subconcussive events’ are referring to impacts that do not result in signs or symptoms of concussion, then presumably ‘concussive events’ refers to those impacts that do result in signs and symptoms of concussion.

One of the problems with the definition of RHI provided in the review [1] is that impact events are described in terms of the outcomes from them, rather than in terms of the biomechanical characteristics of the impacts themselves (i.e. considering the causal agent to be energy transfer). This means that possible interpretations of a ‘concussive event’ for the purposes of researchers trying to quantify exposure to them could include any of the following that an individual sustained:

- an impact event that resulted in having and/or reporting symptoms and/or displaying signs of a concussion injury; or
- an impact event that resulted in a clinical diagnosis of a concussion by a medical professional qualified to provide such a diagnosis; or
- an impact event that resulted in signs or symptoms of brain injury, regardless of the type or severity of brain injury sustained, and regardless of whether medical attention was received or a clinical diagnosis given (noting that not all brain injuries resulting from impact are ‘concussions’—for example, diffuse cerebral swelling, subdural haematomas and other injuries that can result in long-term or permanent disablement or death).

The application of each of the interpretations above would yield different measurements of cumulative exposure to RHI (assuming ‘subconcussive events’ were able to be operationally defined and information about them consistently obtained) and in turn, different relationships with any given health outcome would be apparent, including, in the current case, CTE-NC.

The exposure of RHI is referred to inconsistently in the review article with respect to whether ‘subconcussions’ are included or not. The authors state ‘…these questions also remain for RHI *and* subconcussive impacts…’ [1]. The terminology is further confused in the discussion of criteria for traumatic encephalopathy syndrome (TES) where it is mentioned that ‘all criteria for TES proposed to date, across multiple research groups, require a history of exposure to head injuries, either characterized as RHI, TBI, concussion, or subconcussive injuries’ [1].

In a systematic review of 56 studies looking at subconcussive head impacts in sport, Mainwaring et al. identified that there was no defined minimum threshold for exposure to either ‘subconcussive’ or ‘concussive’ events, concluding that subconcussion was ‘inconsistently used, poorly defined, and misleading’ [39]. They further stated that the terms ‘… “subconcussion” and “subconcussive injuries” are vague and have not been operationalized’ [39].

Nowinski et al. (2024) have reflected on the limitations of the term ‘subconcussive’, calling it a ‘dangerous misnomer’ and noting that a ‘subconcussive’ event does not necessarily involve less force than an event that results in concussion [40]. Their editorial recommends replacement of ‘subconcussive’ with the term ‘non-concussive’ to describe ‘an impact that may be of greater or less force than a concussive impact but is not associated with a diagnosed concussion’ [40]. It remains unclear how the term ‘non-concussive’ would be operationalised and whether a minimum threshold would be applied for a head acceleration event to be deemed a ‘non-concussive impact’. When used in conjunction with ‘concussive impacts’, the term ‘non-concussive’ is still defined by an outcome resulting from the application of forces, rather than in terms of the nature of the forces applied to the head. We believe the term ‘non-concussive’ will suffer from the same drawbacks as ‘subconcussive’ until biomechanical thresholds for such events are developed. Thresholds would need to incorporate the body orientation and posture of an individual at the time the impact occurs in conjunction with the direction and magnitude of force applied.

#### Limitations in Measuring Exposures

Table 1 of the review [1] presents six studies that are heavily relied on to support the argument for a causal relationship between RHI and CTE. The definitions of RHI vary across the six studies and are not comparable to each other. Under the definition of RHIs provided by the authors, exposure to RHIs cannot be quantified, and measurements of the relationship between RHI and CTE-NC—whether causal, correlative or spurious cannot be accurately ascertained, that is, there is no clearly defined causal agent.

**Table 1:** Summary of published cases from case series and case studies as presented in Table 3 of the review [1]

Reference number in original study [Our citation number] Title	Data source and/or case details used in study	Total cases identified as CTE-NC*
VA-BU-CLF brain bank (reference numbers 19, 20, 21, 22)	Reference 22 includes 244 cases, reference 21 includes 223 cases, reference 20 includes 177 cases and reference 19 includes 68 cases.We are unable to differentiate the exact number of unique cases from references 19, 20, 21 and 22, due to overlapping data. Reference 19 precedes the publication of the NINDS/NIBIB criteria	244/223/177/68
References in text (references 23–30)		
Reference 23 [65] Chronic traumatic encephalopathy pathology in a neurodegenerative disorders brain bank	Mayo Clinic brain bank. 4711 cases obtained between 1997 and 2014. Source includes a range of neurodegenerative disorders and a smaller number of neurologically normal controls/list of neuropathologic diagnoses were excluded, leaving 1721 cases, 66 of whom were identified as having history of exposure to contact sport. Those with history of contact sport, plus 66 women and 132 men age- and disease-matched were reviewed for CTE	21
Reference 24 [66] Association of position played and career duration and chronic traumatic encephalopathy at autopsy in elite football and hockey players	Former elite-level American football and ice hockey players selected from a larger brain bank of individuals with pre-mortem histories of mild TBI from different causes; 35 men, 24 former footballers and 11 former hockey players; 29 professional, 6 university or major junior. Other neuropathologies were present in 24 cases (13 co-existent with CTE)	17
Reference 25 [67] Chronic traumatic encephalopathy in Australia: the first three years of the Australian Sports Brain Bank	A total of 21 completed donations to the Australian Sports Brain Bank, 2018–March 2021. All donors had participated in sports with risks of repetitive head injury. All donors had participation history of sports with risk of repetitive head injury; 20 cases had neurodegeneration of some form	12
Reference 26 [68] Chronic traumatic encephalopathy presenting as Alzheimer’s disease in a retired soccer player	Brazil—case study Mixed neuropathological findings of a former professional footballer, aged 83 years	1
Reference 27 [69] Chronic traumatic encephalopathy: a potential late and under recognized consequence of rugby union?	Dublin—case study	1
Reference 28 [70] Tau immunophenotypes in chronic traumatic encephalopathy recapitulate those of ageing and Alzheimer’s disease	Two brain banks: Glasgow TBI Archive or University of Pennsylvania Center for Neurodegenerative Disease Research (CNDR) Brain Bank; 46 cases selected: participation in sport (*n* = 10), TBI from injury (*n* = 4; falls, assault, motor vehicle collision) and various neurodegenerative disorders (*n* = 32). Aim was to look at features of different pathology	4
Reference 29 [71] Mixed pathologies including chronic traumatic encephalopathy account for dementia in retired association football (soccer) players	Queen Square Brain Bank 1980–2010; 16 retired footballers with dementia identified for a clinical surveillance study until death. Subsequently, 14 next of kin gave consent for surveillance and 6 for examination of brains post-mortem	4
Reference 30 [72] Dementia pugilistica: a severe tribute to a career	Algeria, case study 21-year boxing career, several differential diagnoses Precedes the publication of the NINDS/NIBIB criteria	1
Additional references from Table 3		
Reference 67 [73] Histological evidence of chronic traumatic encephalopathy in a large series of neurodegenerative diseases	Total 268 participants (Queen’s Square Brain Bank) Precedes the publication of the NINDS/NIBIB criteria	32 may include cases from ref 29 above
Reference 43 [74] Association between contact sports participation and chronic traumatic encephalopathy: a retrospective cohort study	Mayo Clinic Tissue Registry, 2566 cases between 1 January 2005 and 18 June 2016, 750 with information sufficient for determining sport participation	21 CTE and 21 ‘features of CTE’ appears to overlap with reference 23 above
Reference 74 [75] Detection of astrocytic tau pathology facilitates recognition of chronic traumatic encephalopathy neuropathologic change	Glasgow TBI Archive, aim was to look at features of different pathology; 16 cases	7 but appears to overlap with reference 28 above
Reference 44 [76] Chronic traumatic encephalopathy in the brains of military personnel	The Department of Defense—Uniformed Services University Brain Tissue Repository USA. Study includes 225 brains donated from 2013 to 2021; 217 men and 8 women	10
Reference 75 [77] The delayed neuropathological consequences of traumatic brain injury in a community-based sample	Community-dwelling adults aged 65 and older, without dementia; 532 consecutive participants with brain autopsies (107 with TBI, 425 without)	3
Reference 72 [78] An autopsy proven child onset chronic traumatic encephalopathy	Case study; 36-year-old male with complex medical history from young age (seizures from age 4 and dementia from age 10.)	1

*Not all cases were confirmed with histopathological CTE-NC nor were all confirmed independently of other diagnoses

The use of proxy measures to estimate exposure to a postulated cause is commonplace in epidemiology because it can often be difficult or impossible to obtain actual measures of exposure. Therefore, to develop hypotheses of what relationships ‘might’ hold between exposure to an agent or agents and outcomes, researchers will often use the best estimate of exposure that is available to them.

With respect to RHI in collision sports, proxy estimates have included information from interviews or surveys of participants (or next of kin of decedents) regarding recollections of exposure to brain injuries and time involved in sport participation. Interview and survey data often rely on recollection of events that may have occurred many years previously and are thus subject to information biases including, among others, recall bias, availability bias and unacceptability bias [41–44] (in addition to the direct measurement issues identified earlier).

The impact of information biases is illustrated in the work of Mez et al., who in their Table 1A present data on the number of concussions reported by participants acting on behalf of a decedent examined for CTE-NC [45]. The difference in median concussion count when informants were provided a definition of concussion was remarkable: from a median count of 5 (interquartile range: 1–10) without a definition to 47.5 (IQR: 12–150) when a definition is provided [45]. Further challenging accurate measurement, it is known that participants may choose not to disclose sensitive or personal information, especially if they fear that such information could damage their reputation or have other negative impacts on them if it came to public notice [46]. Researchers have also used participation in contact/collision sports (yes/no), the duration of participation in contact/collision sports (i.e. years played), counts of matches or trainings in which the player was involved during their career and/or the level of play at which the athletes participated as providing proxy measures of exposure to RHI. This information can provide useful insights to understanding potential associations between RHI and health outcomes, but the accuracy of the observed relationship still depends on the degree to which the proxy provides a valid estimate of RHI. The use of participation in collision sports as a proxy for RHIs in studies that have combined a number of sports without controlling for the type of sport played is problematic because the actual exposure to RHI (i.e. the frequency and nature of head impacts) varies widely across activities. Mez et al. acknowledge that ‘years played serves as an imperfect proxy for RHI exposure from American football… an athlete who played for 1 year as a starter on offense and defense may have had more exposure than an athlete who played only sparingly for 1 year’ [45, p 129].

Another method has been to directly measure head accelerations sustained by a sample over a period, and then apply the mean number of head impacts per period from the sample group to the periods of exposure of other groups. This method can yield useful information but it also has well-recognised limitations, such as the fact that the nature and frequency of impacts sustained by participants varies by level of play [47].

The potential influence of confounding variables on observed associations also needs to be considered. Confounding bias is a common problem in epidemiological research and confounding variables need to be accurately measured and accounted for in analyses. Put simply, a confounding variable is an unmeasured, or unaccounted for, factor that is related and has influence on, or from, both the exposure and outcome [48]. Because the unit of epidemiological ﻿﻿research is groups of people, rather than individuals [49], measures applied to groups of athletes such as match involvement and years played in collision sports capture exposure not just to head impacts but many other factors as well. In other words, sports participants, and especially elite/professional athletes, are differentially exposed to a range of factors in comparison to their non-participating counterparts. If those other factors also contribute to later life health outcomes, and they are not explicitly dealt with in the design and analysis of studies, there is a real risk of confounding impacting any observed relationship. Examples of confounders are presented by Iverson et al. in their review of health risks associated with sport-related concussion [50]. The six studies cited in Table 1 of the review [1] either ignore confounding factors or control for only a few common features, such as age and sex.

Information about the validity of proxy measures for estimating exposure to RHIs (however defined) is yet to be published. The limitations in measurement of RHI are important in understanding why many of the arguments presented in the narrative review [1] misrepresent the strength of evidence that currently exists for a potential causal relationship between RHI and CTE-NC.

### Chronic Traumatic Encephalopathy Neuropathologic Change is not a Clearly Defined and Measurable Health Outcome

#### Defining and measuring CTE-NC

Attempts have been made to define the ‘pathognomonic’ lesion of CTE-NC, along with quantification of the extent and distribution of the pathology, so that neuropathologists are reliably able to identify CTE-NC and distinguish it from other pathologies. The concept of CTE-NC is evolving; several descriptions of the defining characteristics of the pathology have been published, with significant differences amongst them [53]. At consensus meetings under the auspices of the National Institute of Neurological Disorders and Stroke (NINDS) and National Institute of Biomedical Imaging and Bioengineering (NIBIB), a required feature of the proposed 2016 article definition [phosphorylated tau (p-tau) aggregates within astrocytes] [52] was dropped in the updated definition of 2021, while an additional nuance—the depth of p-tau aggregates relative to the pial surface—was added [51]. Essentially, what was counted as a case in a study published a decade ago would not necessarily be counted as a case were the study to be done today. Such changes to the definition of CTE-NC can result in substantial differences to estimates of prevalence and observed associations with putative causal factors over time. For example, 98% (117 of 119) of professional football players were reported to have CTE-NC in a study from the Boston University CTE Center’s brain bank in 2017 [54], whereas a 2023 study from the same brain bank that included an additional 165 professional players, and which reported that the 2021 consensus definition had been used, found CTE-NC in 251 of 284 cases (88%). It is unclear from the paper whether the retroactive application of the 2021 criteria resulted in cases being reclassified from ‘CTE-NC’ to ‘no CTE-NC’ (assuming that the criteria were applied across the entire case-series). If the new criteria were not applied retroactively then there would be different thresholds for cases depending on the time at which the case was evaluated. The application of the new criteria appears to have coincided with a decrease in the percentage of professional football players diagnosed with CTE-NC.

The NINDS/NIBIB consensus group in 2021 ‘endorsed a single pathognomonic lesion in the cortex as the minimum threshold for CTE’ and further suggested additional bilateral sampling under certain circumstances including ‘clinical concern’ [51]. The low threshold, together with extensive tissue interrogation, results in maximum sensitivity towards a (statistically) positive case outcome. Maximising sensitivity comes at a cost to specificity: in this case, the plausibility that such a minimal neuropathological outcome has any clinical relevance. In other words, even if a causal relationship between RHI and CTE-NC using such definitions were demonstrated, any biological or clinical significance would remain an open question. This vital issue is not addressed in the article of focus [1].

Once﻿ defined, a health outcome in epidemiology (in this case CTE-NC) also requires that a valid and reliable measure of the outcome can be used by researchers. Throughout the review [1] is the assertion that the cited evidence examines neuropathologically ‘confirmed’ cases, largely from the US Department of Veterans Affairs—Boston University—Concussion Legacy Foundation (VA-BU-CLF) brain bank. This is problematic because pathologists can and do disagree on the presence and extent of CTE neuropathology [52]. Distinguishing CTE neuropathology from concomitant neurodegenerative and ageing-related pathologies is currently a significant challenge for diagnosticians. More information is provided in Additional File 2—Threshold and measurement of CTE-NC.

#### Clinical syndromes linked with CTE-NC

Traumatic encephalopathy syndrome (TES) is a provisional research construct that was initially proposed in 2014 as an attempt to identify clinical correlates with CTE-NC at autopsy [55]. The original criteria were heavily focussed on mental health problems, and concerns were raised about whether they could be used to reliably distinguish between individuals with CTE-NC and those with other conditions [56]. For example, Mez et al. [57] reported on 309 donors to the VA-BU-CLF brain bank, of which 244 had CTE. With the pathological diagnosis of CTE-NC as the gold standard, the clinical diagnosis of TES demonstrated a sensitivity of 0.97 and a specificity of 0.21. Interpretation of these statistics indicates that the 2014 clinical criteria for a diagnosis of TES are very sensitive (i.e. few cases would be missed), but the specificity is very poor (i.e. the 2014 criteria for TES did not provide clinicians with a decision support process from which to distinguish CTE from other conditions that affect mood, behaviour and cognition).

In 2021, TES was redefined by a group of clinicians and researchers using a modified Delphi process [58]. Psychiatric features, such as ‘anxiety, depression, apathy, and paranoia’, which were considered core clinical features in the research criteria proposed for TES in 2014, were moved from ‘core’ to ‘supporting’ features. Despite the change, the question of specificity persists with the new criteria. In a sample of 507 older adults evaluated by Terry et al., approximately 1 in 4 met the symptom criteria for TES, many of whom had no history of repetitive neurotrauma [59]. Terry et al. also surveyed 1100 participants from a national health volunteer registry using the refined TES criteria, again finding that it was not possible to distinguish symptoms related to repeated head trauma or concussion from other conditions, particularly mental health related conditions, in the general population [60]. Similarly, Iverson et al. compared brain donors within the VA–BU–CLF brain bank, and found no statistically significant differences in any of 11 mental health outcomes in those with CTE-NC at autopsy compared with those without CTE-NC [61].

Given the lack of specificity, the current TES research criteria do not appear to provide a basis for clinical appraisal, and the authors of the criteria state: ‘These NINDS Consensus Diagnostic Criteria for TES are meant primarily for research purposes and should be used cautiously in clinical and medicolegal settings, avoiding equivalence with a diagnosis of CTE, and using appropriate care when communicating a diagnosis of TES’ [58, p 860].

## Testing Causal Hypotheses is Premature

Bradford Hill’s considerations were developed under the assumption that there are results available from hypothesis-testing studies, such as case–control and cohort studies. To the best of our knowledge, findings of studies using such designs to examine whether RHIs cause CTE-NC, and whether CTE-NC represents a progressive neurodegenerative disease, have not yet been published.

Beyond the fact that the conditions for causal claims are yet to be met, we believe the statements by the authors [1] implying that the application of the Bradford Hill considerations means that RHI has been established as the cause of CTE contains several significant inaccuracies. In the sections below we provide our reasoning for our belief, and highlight research methods and approaches to public health and risk management that we think can better address the concerns raised by the authors [1].

### Strength of Association

The strength of association among variables is often presented via statistics such as correlations, risk and rate differences and hazard, risk, rate and odds ratios. While there is a general premise that a stronger (positive) association between an exposure and outcome is more likely to be a relationship that is causal in nature, this does not always hold:

*…a strong association is neither necessary nor sufficient for causality…* [62]

The review authors describe ‘six well-conducted case–control studies where the researchers made a reasonable attempt to identify RHI history and had more than 50 subjects to be sufficiently powered for statistical significance…’ [1, p 4]. The data available in the six studies provide either weak evidence, due to a mix of ascertainment bias and unclear validity of proxy measures used to estimate exposure to RHI, or are unsuitable to report odds ratios reflective of the likelihood of developing CTE-NC on the basis of exposure to RHI due to the way cases and controls were selected. We agree with the authors of a systematic review that these six studies are not case–control or cohort designs [50].

Because these studies do not meet the requirements of a case–control design, the odds ratios presented are invalid. As an example, one of the key sources is the VA–BU–CLF brain bank, which sought inclusion of cases on the basis of their exposure to RHI in the first place.

*All 269 brains from the VA–BU–CLF Brain Bank had contact-sports history.* [1]

If cases are selected into a case–control study *because *they were exposed, the odds of exposure in the case group are meaningless. In turn, the odds ratio comparing exposure in the case group with that of the controls does not represent the relationship between development of the outcome and exposure to the agent. The selection of cases on the basis of exposure is why some of the calculations reported required imputed values, and why the odds ratios appear unusually large. In addition, because the prevalence of CTE-NC is so high in the VA–BU–CLF brain bank, applying the rare disease assumption to the use of odds ratios to approximate relative risks is inappropriate, and yields a gross overestimate of the risks [63]. Refer to Additional files 4: Case control studies and odds ratios.

### Consistency

Consistency refers to repeated findings within and between studies, settings, timepoints and populations, and similar to strength of association, consistency does not necessarily imply a causal relationship, nor does lack of consistency rule out a causal association [62]. In their Table 3, the review authors present ‘CTE cases diagnosed globally’ as justification for causality through consistency, with a summary of case series published by different brain banks/groups [1]. The consistency of brain bank data demonstrating both CTE pathology together with a retrospective history of RHI, regardless of whether co-morbid pathology is present, is difficult to accept as evidence for causation, given the sources of error with outcomes and exposures already described.

‘McKee et al. (reference number 18 [64],) noted that prior to 2009, there were only 48 cases of CTE in the literature, in contrast to the hundreds of cases of CTE since (reference numbers 19–30)’ [1, p 2]. Further, in their Table 3, the authors present ‘the largest CTE case series’ published at various brain banks that are ‘understood to be using NINDS/NIBIB consensus criteria for diagnosis’ [1, p 5–6].

These data (from references 19–30 and Table 3) on the number of CTE cases diagnosed globally are presented as justification for causality through consistency. There are overlapping cases from the VA–BU–CLF brain bank (reference numbers 19, 20, 21, 22) and we are unable to differentiate the exact number of unique cases from these series. Table 1 summarises the sources cited, noting critically that not all cases in this table are confirmed with histopathological CTE-NC. Some findings were based on the first NINDS/NIBIB consensus meeting (published in 2016), some cases have multiple diagnoses, and two studies were published in 2015, before the first NINDS/NIBIB criteria were reported.

We emphasise that generalisations and exaggeration are not helpful for understanding the natural history or pathology of CTE-NC and any potential relationship with RHI. The information further empasises the importance of defining and measureing exposures and outcomes accurately.

### Specificity

*RHI is the only factor common to reported CTE cases, and there is almost no evidence of CTE in those examined who have not sustained RHI* [1, p 7].

We believe the claims made by the review authors [1] in support of this section to be misleading of the current literature, particularly as they chose to focus on RHI as the lead risk factor for CTE-NC, as opposed to exploring the question ‘what are the potential contributing risk factors?’.

There are several alternative, and reasonable, factors explored in the literature that may cause CTE-NC, either independently of, or in conjunction with, RHI, including genetics, inflammatory responses, ergogenic aids and substance use to name a few [79, 80]. There is also more than ‘almost no evidence’ of CTE-NC without RHI, with various cases from literature having been described [81, 82]. Finally, irrespective of the issues identified, and sufficient alone, is that the absence of evidence is not evidence for its absence. Rather, we need to continue asking the right questions and addressing them with suitable study designs.

### Temporality

Temporality is perhaps one of the more intuitive concepts to understand in establishing cause: the risk factor (or exposure) must occur before the disease (or outcome). While temporality seems straightforward, until there are clear parameters to define and measure RHI and better information regarding any clinical manifestation of CTE-NC is available, this viewpoint also remains uncertain.

Establishing temporal associations of RHI and CTE-NC is challenged by not knowing the evolution of CTE-NC before, during and after RHI exposure, whether any pathology becomes stationary, whether it is reversible, or whether it is progressive (and why that might be the case in the absence of further exposure to RHI) [83]. The authors write:

*As outlined in the above section on specificity, the exposure to RHI is associated with CTE pathology and, especially with the introduction of the aforementioned revised NINDS/NIBIB neuropathologic criteria requiring neuronal involvement in the perivascular deposition of tau, this pathology occurs nearly exclusively in the presence of clearly identified RHI exposure* [1, p 7].

Here, the authors have exemplified several of our concerns with the claim of ‘RHI’ being a ‘clearly identified exposure’, as presented earlier.

### Biologic Gradient (dose–response)

This section of the causal claims rests partly on a description of historical cases. The condition termed ‘CTE’ in reports of ‘dementia pugilistica’ among boxers is qualitatively different from that which is currently termed CTE-NC. This is incorrect and misleading of what is currently known about CTE-NC.

The following two quotes exemplify how the authors of the review equate ‘dementia puglistica’, or ‘punch drunk’ syndrome, with modern conceptions of CTE:

*Dr. Harrison Martland is credited with first identifying the syndrome that was later called CTE in his article Punch Drunk, published in the Journal of the American Medical Association in 1928* [1, p 7].

*While CTE has been known in the literature for nearly a century, most of the research on CTE has occurred only in the last decade* [1, p 2].

These statements leave the reader with an impression that historical punch-drunk syndrome and modern day CTE are exchangeable when, in fact, they are markedly different. Punch-drunk syndrome, characterised by clinical signs such as dysarthria, shuffling gait and Parkinson’s-type symptoms, was identified and conceptualised on the basis of clinical neurological examination (multiple and variable neurological deficits from extreme neurotrauma exposure). Modern CTE-NC is a purely neuropathological finding (or more specifically, an immunohistochemical finding) that, thus far, lacks a specific clinical presentation. Neither Martland nor Critchley reported pathological changes as is implied. Further, the work of Goldfinger et al., who re-examined the Corsellis series using the 2016 NINDS/NIBIB criteria and modern immunohistochemical techniques, refuting several of the original Corsellis findings, has been overlooked in the narrative review [84].

Focal deficits attributed to boxing such as slurring dysarthria, tremor and gait disturbances at or before retirement, were common in early twentieth-century boxers with prolonged neurotrauma exposure. These visible signs are not commonly seen in the case of modern athletes; for example, one is hard pressed to find even a single case of an American football player with a focal neurological deficit at retirement. Further information is presented in Additional files 3—Misrepresentations of historical research*.*

The issue of selection bias is also raised in this section on biological gradient. Selection bias describes a systematic difference in the relationship of exposure and disease between those who participate in a study and those who in theory could be eligible for the study but did not partake [85]. In the case of brain bank cohorts, participants are not randomly assigned to be investigated, rather they or their next of kin choose to donate their brain, often because of specific health concerns. Because the donation of brains into many brain banks is based on symptoms during life, as well as contact sport or RHI exposure, apparent relationships found in the data may not generalise to the wider populations.

To account for selection bias, the authors [1] refer to the findings of LeClair et al. [86], who explored the influence of selection bias through simulated analyses. While the use of quantitative methods of bias analysis is endorsed by experts in epidemiological statistics, such as Greenland [87] and Lash et al. [88], both highlight that the methods often require the use of unverifiable assumptions about probabilities of selection and non-selection across groups. To the extent that modelling does incorporate such assumptions, the results of sensitivity analyses reflect plausible conjectures about the effects that would have been found had selection bias not been a feature of the study, rather than direct evidence of the size and direction of the true effect. As Lash notes, ‘…bias analyses do not establish the existence or absence of causal effects any more than do conventional analyses’ and ‘…when examining a bias analysis, a reader must bear in mind that other reasonable inputs might produce quite different results’ [88, p 714].

In relation to the LeClair study, Nowsinki et al. write ‘the researchers found that highest level of football play was associated with CTE diagnosis in a dose–response manner’ [1, p 9]. However, as described in the study limitations by LeClair et al., ‘exposure’ was treated as a categorical variable in which the ‘…highest level of American football playing served as a proxy measure for RHI…’ and ‘we were unable to consider other measures of exposure, such as frequency of RHI, or even duration of play…’ [86, p 1441]. Ultimately, the ‘dose’ argument of RHI has shifted well away from being ‘the cumulative exposure to recurrent concussive or subconcussive events’ [1, p 2].

### Plausibility

Findings from animal and simulation studies are provided in the narrative review as examples that ‘provide evidence of a credible mechanistic hypothesis for the location of the pathognomonic lesion and the association between RHI and CTE alongside the paucity of CTE cases in individuals not exposed to RHI all support that RHI exposure is a plausible cause of CTE’ [1, p 9]. We accept that the evidence presented in this section is consistent with the hypothesis that RHIs may be a causal factor in CTE-NC. Further, we recognise the value that animal studies and simulations have in understanding the aetiology of human disease processes, challenges in translating findings from animals to humans notwithstanding. As Shimonovich et al. [24] write, however: ‘the plausibility of the causal relationship is both dependent on and limited by knowledge available at the time. It may be further limited by assumptions based on investigators’ beliefs rather than empirical evidence’ [24, p 882].

### Coherence

Coherence requires that what is known about the cause and effect proposed does not conflict with what is known about the natural history of disease. The review authors state ‘we must demonstrate that the association between RHI and CTE pathology does not conflict with what we know about the development of CTE pathology or RHI’ [1, p 9]. As pointed out elsewhere, however, coherence, ‘provides at best only weak support for causality, because many theories will exhibit such coherence, including most theories that are proposed and eventually refuted’ [89, p 21].

In their section on coherence, the authors [1] briefly consider the potential of other causal variables. The authors focus on opiate misuse as the only variable that ‘has been proposed as a potential alternative cause of CTE’, subsequently dismissing it on the basis of one study that reported ‘tau deposition from opiate use is easily distinguished from the pathognomonic CTE lesion’. They conclude the section with the following statement: ‘With what is known in the literature about computer modeling of brain trauma, post-mortem confirmed cases without a history of RHI exposure, sex differences, opiate use, and CTE genetics, RHI remains the only candidate risk factor for CTE causation’ [1, p 10].

We recommend further scrutiny of existing evidence before drawing conclusions from these arguments of coherence, not least because studies that would permit proper evaluation of a range of possible contributing factors to the development of CTE have not yet been conducted. We note that other authors have raised multiple candidate risk factors for both CTE-NC and clinical and functional outcomes, including pre-existing psychiatric conditions, sleep disorders, substance use, chronic pain, genetic factors and exposure to anaesthesia [79, 90]. We do not believe that the statement that RHI is the only candidate risk factor for CTE causation is well supported by the evidence at this point.

### Experimental Evidence

Although all study designs used in epidemiology have their limitations [87], findings from well-designed cohort studies and case–control studies are generally accepted among epidemiologists as capable of testing causal hypotheses. Randomised controlled trials are best suited to testing causal hypotheses but are limited in their application for many public health issues, including injury. Clinical case-series and cross-sectional studies have important roles in epidemiology, especially with respect to identifying novel health outcomes and developing hypotheses to be tested in more rigorous designs. Case-series and cross-sectional studies also have significant, and well-recognised, limitations with respect to generalising results from the individuals and groups studied to the wider population.

Establishing causation (or not, as the case may be) between RHI and CTE-NC can in principle be ascertained via the application of observational research designs [91]. Such studies need to be designed to properly account for the effects of random error, confounding, information bias and selection bias. Prospective and retrospective cohort studies, as well as case–control studies, could be developed that would provide answers to many important questions including:

- whether, and to what extent, RHIs are a causal factor of CTE-NC;
- whether, and to what extent, factors other than RHIs cause CTE-NC;
- whether CTE-NC represents a progressive neurological disease; and
- whether, and to what extent, CTE-NC causes the range of clinical outcomes to which it has been linked via cross-sectional analyses.

The same or similar studies [92] could simultaneously address the effects of RHI on other health outcomes of interest, such as depression, neurodegenerative diseases and dementia, and whether they were related to CTE-NC.

Once definitions of RHIs and CTE-NC are developed, agreed on and validated, cohort (retrospective and prospective) and case–control studies are likely to provide much stronger evidence of the relationship between RHIs and CTE-NC than has yet been presented, at which point cautious judgements about the likelihood of observed relationships being causal can, and should, be made. In their discussion section, the authors have implied such studies are impossible to conduct, with the claim that they would require unfeasible studies of identical twins and unethical assignment to groups which are, and are not, subjected to head injuries from early in life. We disagree with that view of study design and reiterate that much research in public health is conducted using observational designs, with true experimental designs unsuitable in many public health scenarios [93].

### Analogy

In this section, analogies are drawn between the level of evidence that was obtained regarding the causal relationship between cigarette smoking and lung cancer being ‘well established’ with that between RHI and CTE-NC, and further between issues regarding how exposure to cigarette smoke has been quantified in observational studies and how exposure to RHIs have been quantified.

In their review [1, p 11], the authors claim that with respect to exposure to cigarette smoking ‘key questions remain unanswered or incompletely answered, including what precisely constitutes a smoked cigarette (the dose), why some smokers develop cancer and others do not, how many cigarettes are too many, or which specific cigarette or carcinogen sparked the lung cancer’ [1, p 11]. No cited evidence is provided in support of their claim that the lack of a precise measurement of a smoked cigarette is actually a feature of the epidemiological evidence, but they do use it to set up the following argument: ‘The fact that these questions also remain for RHI and subconcussive impacts is often raised as a reason that conclusions on RHI/CTE causation cannot be drawn’ [1, p 11]. They conclude that: ‘These knowledge gaps have not limited the ability to assert a causal link between smoking and lung cancer, and similarly should not limit the ability to determine the likelihood of a causal link between RHI and CTE’ [1, p 12].

The argument is an example of the ‘straw man’ fallacy. Focussing on the first claim, regarding exposure to smoked cigarettes, a systematic review and meta-analysis examining survey-based assessments of exposure to cigarette smoking published in 1994 found that self-reported smoking status had generally high levels of sensitivity (87%) and specificity (89%) when validated against biochemical measures of exposure across the 26 studies [94]. Although it is acknowledged that survey methods provide less accurate information in some situations (for example, when assaying cigarette use among pregnant women), [94] there is no doubt in the epidemiological community that measures of ‘dose’ captured through surveys asking about cigarettes smoked per day or pack years of exposure have yielded valid information regarding the link between smoking and lung cancer.

The qualitative and quantitative differences in the amount of evidence regarding smoking causing lung cancer and RHIs causing CTE-NC are currently so large that claims of the two issues being comparable are misleading. As noted above, the studies identified in the review [1] regarding the relationship between RHI and CTE-NC have employed a range of approaches that have yet to be validated in assessing exposure to RHIs, along with definitions of CTE-NC that have varied over time. To date, studies have primarily used case-series and cross-sectional designs with papers from overlapping subsets of the VA–BU–CLF brain bank case series providing the data for the great majority of the existing publications, as well as the case material for consensus efforts. The autopsy case-series data have been supplemented by interviews and surveys of ‘informants’, who are predominantly next of kin of the deceased.

By contrast, the use of case–control and cohort designs using consistent methods of appraising exposure are a feature of the studies examining the relationship between smoking and lung cancer in humans. The amount of supporting evidence for the contention that cigarette smoking causes lung cancer differs from that regarding RHIs and CTE-NC by an order of magnitude. A systematic review and meta-analysis published in 2012 of the results of studies published up till the year 2000 identified 267 ‘principal’ (and 20 subsidiary) studies, of which 209 used case–control and 52 used prospective cohort designs [95].

Questions such as ‘which specific cigarette sparked the lung cancer’ or ‘which specific head impact sparked the development of CTE-NC’ might form the basis of legal arguments or judgements regarding insurance claims but are not questions that epidemiological studies would address because the questions are directed at individuals, not at population groups. The question of why some smokers develop lung cancer while others do not is relevant to considerations of how cause is conceptualised, but as discussed by Brand and Finkel [96] and reiterated in the review [1], the fact that a cause of a health outcome is a cause does not necessarily imply that all those exposed to it will develop the outcome, nor that people not exposed to it will not. It does, however, imply that factors other than the specified cause contribute to the outcome.

Cohort study designs, which permit the evaluation of multiple potential mediators and confounding variables are valuable in enabling understanding of how strongly associated with an outcome a given cause is, and how that cause interacts with other potential causes and confounding factors. The claim that RHIs are ‘the only candidate risk factor for CTE causation’ [1, p 10] reflects a lack of information about other candidate risk factors derived from studies that would provide good evidence about them, rather than reflecting supporting evidence for the contention that RHIs are, in fact, the only candidate risk factor.

### Comments on Children’s Participation in Sport

In the discussion section of the review, the authors emphasise the question: what impact do head injuries sustained in youth sport have on participants in the long term? writing*:*

*Perhaps most consequential would be the positive health impact for children. As it stands today, tens of millions of children as young as 5 years old are exposed to RHI in sports because they are playing by rules that were originally designed for adults. Armed with confidence in the causal connection between RHI and CTE, parents and youth coaches may reject exposing their children to a preventable degenerative brain disease simply because the current rules (tackling, heading) make RHI inevitable, especially when non-RHI versions of those contact sports exist, as well as alternative sports without RHI. Considering that both CTE onset and severity have been associated with a dose-response, strict reforms that lower the dose could effectively prevent new cases of the disease* [1, p 13–14].

This addition to the paper doesn’t follow from the prior content, because evidence about the effects of children’s sport on health outcomes was not presented. Rather, it serves as an appeal to inherent emotional concerns. The statement was made upfront that ‘any reference to CTE in this review refers to cases that have been confirmed by autopsy’ [1, p 3] so declarations that ‘parents and youth coaches may reject exposing their children to a preventable degenerative brain disease’ [1, p 14] is overreaching.

In a 2019 narrative review into the age of first exposure to tackle football and later life outcomes Alosco and Stern stated ‘…it is our opinion that more methodologically rigorous research on the long-term neurologic consequences of youth tackle football is needed before policy and safety guidelines can be accurately informed’ [97, p 113]. Further, in a 2021 narrative review, Iverson et al. [98, p 1] concluded ‘The accumulated research to date suggests that earlier AFE (age of first exposure) to contact/collision sports is not associated with worse cognitive functioning or mental health in (i) current high school athletes, (ii) current collegiate athletes, or (iii) middle-aged men who played high school football. The literature on former NFL players is mixed and does not, at present, clearly support the theory that exposure to tackle football before age 12 is associated with later in life cognitive impairment or mental health problems’. More recently, in 2023, the Concussion in Sport Group did not find evidence to support the notion that participants in youth, high school and collegiate sports are at risk for long-term consequences [50].

Of course, encouraging individuals and sports organisations to reduce exposure to brain injuries is sensible, and sports organisations have made relevant changes that are being closely monitored. For example, the age for body-checking in youth ice hockey was raised [99–102]. There has also been a general cultural shift to understand reasoning for, and discourage, poor compliance from players, parents or others with changes that are implemented to reduce exposure to, and consequences from, brain injuries sustained in sports [103, 104]. The review authors [1] call out the ‘tens of millions’ of children who have participated in contact or collision sports during their youth and suggest they have higher risks of developing neurodegenerative diseases later in life than those who have not played such sports. There is no basis for this claim presented in their review. Such sentiment needs to be avoided to do justice to the importance of this issue and to respect those whose health has been impacted by brain injuries sustained during their participation in sport.

## Discussion

We raise the counterpoints to the authors’ [1] interpretation of the Bradford Hill considerations because the paper has had significant influence and the issue is important. The paper has been repeatedly promoted as having definitively established causality between exposure to RHI and CTE [3]. This claim is not supported by the arguments presented in the paper, nor by systematic appraisals of the wider evidence [50].

There are sound reasons why systematic reviews and meta-analyses are preferred over narrative reviews when researchers seek to evaluate questions of causal relationships. Published guidance on how to conduct systematic reviews and meta-analyses of observational studies (e.g. COSMOS-EA) [105] and how to evaluate a given corpus of evidence (e.g. GRADE) stress the importance of having a pre-defined and well-documented search strategy, clear criteria upon which studies are included or excluded and explicit evaluation of study design and biases [106]. The aim of the systematic process is to ensure that the entirety of the evidence is properly, and without bias, evaluated, synthesised and summarised to produce the best evidence base to inform clinical and health policy.

As creators and consumers of research, it is up to all of us to question findings and approach their interpretation with caution and critique. The question of whether RHI causes CTE-NC remains open for two reasons. The first is that the absence of clear operational definitions of postulated causal agents and health outcomes means that exposure to the agent cannot be accurately quantified, and thus health outcomes cannot be accurately related to exposure to the potential causes. This fact currently represents an undercutting defeater of any causal claims between RHIs and CTE-NC, because those minimum requirements have yet to be met. Meeting them requires the development of operational definitions of RHIs and CTE-NC that become generally accepted by the research community, and consistently applied in future studies. Secondly, even if scientific consensus on what constitutes RHI and CTE-NC had already been established, the application of those operational definitions in the studies from which the review authors draw their conclusions would still not be sufficient to make any causal conclusions on this matter due to the inherent limitations in case-series and cross-sectional study designs. Rather than overstating the implications of hypothesis-generating studies, we could better move this debate forward by undertaking hypothesis-testing research designs such as cohort and case–control studies.

Although the review authors [1] do not ‘substantially explore the separate question of a causal relationship between CTE neuropathology and clinical symptoms’, that question is arguably of greater public health relevance than the question of a causal relationship between RHI and CTE-NC. Understanding whether findings of CTE-NC at autopsy represents an important public health issue depends on both how prevalent CTE-NC pathology is in various populations, and how strongly related CTE-NC is to clinical syndromes, neither of which are yet established.

In a systematic review of the long-term consequences of sports concussions, Iverson et al. did not find any case–control or cohort studies addressing the risk of CTE-NC after head injury or participation in collision sports [50]. Other authors have also highlighted a lack of hypothesis-testing study designs compounded by high-volume publication of hypothesis-generating studies in the TBI literature [107–109]. Iverson et al. [50] also reported on several studies comparing professional athletes with the general population that found associations between collision sports participation and dementia and amyotrophic lateral sclerosis (ALS) as a cause of death. In the case of American football, for example, a small fraction of participants compete professionally. For the remaining majority of amateur American football players, there is no literature to indicate any adverse long-term neurological or psychiatric problems from contact sport participation [50]. Moreover, studies suggesting ‘neurodegenerative’ associations in professional athletes are dominated by ecological designs (e.g. studies of death certificates spanning several or more decades with data mining of health records) that lack individual exposure data and adequate control for confounding (e.g. genetics, demographic, health related or environmental) [110, 111] make it difficult to infer risk, let alone cause. Other similarly designed studies showed no associations with neurological, including neurodegenerative, problems [112, 113].

We strongly encourage researchers to undertake studies to address the gap in knowledge for contributing factors of CTE-NC and note that several are now in progress (selection of examples cited [114–118]), along with further investigation of the relationships among CTE-NC, co-existing pathologies and clinical outcomes.

There is an assumption in the review [1], as well in as other research on CTE [119], that CTE-NC represents a canonical neurodegenerative disease, and that it causes clinical outcomes, for which the evidence available is lacking. The question as to whether CTE-NC or some other pathological outcome yet to be elucidated is related to clinical signs and symptoms among individuals exposed to brain trauma is a high-priority question still to be answered. With that being said, a responsible approach to managing risks to participants in collision sports is to utilise the precautionary principle, and for sports administrators, regulators and other interested parties it is to act to eliminate and minimise the frequency and magnitude head impacts as far as is reasonably practicable, and to educate participants about prevention and avoiding injuries in sports. In our view, this needs to happen even though considerable uncertainty about the presence and magnitude of the risk across different sports remain. Importantly, managing risks in sport does not imply eliminating all injury risks [120], whether injury to the brain or otherwise.

## Conclusions

This evaluation of evidence presented in a review article [1] identified several inaccuracies and misrepresentations that refute claims of a “definitively established” causal relationship between RHI and CTE-NC. The fundamental criteria for establishing causality are not fulfilled. We have identified that the quantity and quality of the evidence in the review does not support the conclusions the paper draws and that the discussion and conclusions sections are a series of arguments advocating for acceptance of their claims, rather than offering a rigorous scientific evaluation of the evidence presented to substantiate those claims. Alongside methodological work to establish clearly defined and quantifiable variables, the conduct of well-designed cohort studies, with attention on a wide range of candidate risk and protective factors, are in progress. Until the findings of several such studies are published, the scientific community, and all those who distribute research findings, must be cautious of making or accepting causal claims in this field.

## Supplementary Information

Below is the link to the electronic supplementary material.

Supplementary file1 (PDF 306 kb)
