# Supplementary material for: Epidemiological Principles in Claims of Causality: An Enquiry into Repetitive Head Impacts (RHI) and Chronic Traumatic Encephalopathy (CTE)
Source: Sports Med. 2024 Sep 15;55(2):255–74. doi: 10.1007/s40279-024-02102-4 (PMC11947058; doi:10.1007/s40279-024-02102-4)
Supplement: Supplementary file 1 — Supplementary file1 (PDF 306 kb) [file 40279_2024_2102_MOESM1_ESM.pdf]

doi 10.1007/s40279-024-02102-4

Journal: Sports Medicine

Epidemiological principles in claims of causality: an enquiry into repetitive head impacts (RHI) and chronic traumatic encephalopathy (CTE)

Lauren V Fortington[1]*, J David Cassidy[2], Rudolph J Castellani[3], Andrew J Gardner[4], Andrew S McIntosh[5], Michael Austen[6], Zachary Yukio Kerr[7], Kenneth L Quarrie[8-10].

*** Correspondence:**
Lauren Fortington
lauren.fortington@gmail.com

doi 10.1007/s40279-024-02102-4

Additional file 1: Introduction to head impact forces

A head impact is a very short duration event, often less than 10 milliseconds, during which a force is applied to the head. The source of the impact force can be direct contact with the head or contact with other part(s) of the body that results in forces applied via the cervical spine (indirect). A head impact can have a range of consequences from minimal to catastrophic because the impact can distort the skull, brain, and vascular structures resulting in tissue failure.

Largely because of measurement reasons, head impacts are most often characterised by the head's response to the impact, e.g., linear, and angular accelerations, rather than the force(s) applied to the head. The linear acceleration of the head is typically reported as multiples of one gravity (9.81 m/s^2) and can range from as low as approximately 2 g's (e.g., during foot contact in running) to greater than 1,000 g's in a head impact at 7.7 m/s against a rigid surface (e.g., a fall from height onto concrete).

During foot contact, jumping and landing, and some 'whiplash' type events, the head impact is considered as indirect because the impact force is transferred to the head from the body via the neck. In many instances in sport, the workplace, recreation, and motor vehicle crashes, a direct impact occurs in which the head is stationary and struck by a moving object or is moving and strikes another object (moving or stationary). If we consider peak linear head acceleration as a marker for head impact severity, the following general associations can be seen between peak linear head acceleration and events or outcomes (**Additional files Table 1**.)

Additional files Table 1. Peak linear head accelerations recorded for common sports activities, derived from [1–8]

Peak linear head acceleration	Example of activity	Usual outcome
0 to 10 g	Indirect impacts: e.g., vigorous physical activities such as running, jumping and landing; light heading in soccer/football.	No injury
10 to 20 g	Direct impacts: e.g., heading in soccer/football.<br>Indirect impacts: e.g., shoulder collisions; heading in soccer/football.	No injury
20 to 30 g	Direct impacts: e.g., re-directional and finishing headers in soccer/football; incidental direct head contact in sport.	Possible superficial injury and/or discomfort
30 to 60 g	Clearly observable direct head contact: e.g., 'light' boxing punch to protected head; shoulder impact to head; fall to ground when tackled.	Superficial injury, pain/discomfort, concussion possible in upper range
60 to 100 g	Direct head contact: e.g., 'light' boxing punch to unprotected head; low fall from bicycle with protected head (bicycle helmet); clearly observed shoulder-to-head; fall from jump to ground; head-to-head impact. Cases of indirect head impacts are rare but can occur in tackles to the body that cause rapid acceleration to the head via the neck.	Range of concussion in sport.
100 to 200 g	Direct impacts: e.g., 'heavy' boxing punch to unprotected head; up to 1.5m @ 25 km/h fall from bicycle with protected head (bicycle helmet); high running speed head-to-head impact in sport.	Mild to moderate TBI.
200 to 300 g	Direct impacts: short fall (0.5 m) from bicycle with unprotected head; projectile impacts (cricket ball, ice hockey puck, baseball) to unprotected head.	Serious to severe TBI including cranial fractures and intracranial haemorrhage

Note: g: gravitational constant; TBI: Traumatic Brain Injury; Reference sources for table: [1–8]

Additional file 2. Threshold and measurement of CTE-NC

Considerable efforts have been undertaken in defining a diagnostic pattern of p-tau labelling and pinpointing CTE-NC as a distinct tauopathy. For balance, however, the reader should be aware that neuropathologists can and do disagree on CTE-NC in individual cases and even individual p-tau patterns on the same pathology slide. The Cohen's kappa in blinded review was 0.67 (10/25 were CTE cases) in the 2016 consensus effort [9] and 0.63 (17/27 were CTE cases) in the 2021 consensus effort [10]. In the 2016 consensus article [9] some reviewers (neuropathologists with specific expertise in tauopathies) diagnosed pathologies other than CTE in presumptive CTE cases, and some reviewers diagnosed CTE in presumptive non-CTE tauopathies. At least one expert diagnosed CTE in 8 out of 15 non-CTE cases, while in one presumptive non-CTE tauopathy, CTE was diagnosed by 5 out 7 experts. Such uncertainties are not unexpected in a complex and evolving field, but the diagnostic reliability challenges are important to acknowledge in discussions of aetiology.

The low threshold for CTE-NC (set by a "group endorsement") may not be fully appreciated by those without a background in neuropathology. Such p-tau immunolabeling might occupy a microscopic field of less than 1 millimetre in diameter, while the underlying structure of the tissue in that field is entirely normal by routine histopathology. There is no loss of neurons, no reactive gliosis, and no change indicating that some form of "damage" took place. A "single pathognomonic lesion" is a microscopic colour change visible with the application of reagents, similar to scant p-tau labelling in other brain regions that appear as a variation of normal as early as childhood [11]. The numerous cases in the literature reported as "low-stage," notwithstanding the unreliability of staging, fall within this extremely small range of p-tau staining, and we argue that it is implausible that such labelling would be strongly associated with clinical outcomes, especially given the lack of such relationships observed in pre-mortem function and post-mortem neuropathology found in some other neurodegenerative diseases [12,13]. This is supported by recent studies in young athletes that showed no correlation between CTE-NC and any clinical problem [14], and a large scale clinicopathological validation, in which there was no difference between any of a number of mental health outcomes in those with and without CTE-NC at autopsy [15,16]. Iverson et al further note that there is presently no study indicating increased risk of psychiatric problems as a long-term consequence of sport at any level, whereas some studies showed a decreased risk [17]. The question therefore of causal relationship between RHI and CTE-NC as currently defined, which remains open, may be inconsequential pending further refinements in both parameters, as the exposure is ill-defined and the (immunohistochemical) outcome appears to fall below the threshold of clinical detectability.

The extent to which presumably larger amounts of CTE-NC conflate with Alzheimer's disease-related p-tau and aging-related p-tau also remains an ongoing discussion. The 2021 consensus group stated "*Distinguishing CTE from concomitant neurodegenerative and age-related pathologies represents a topic of interest for future studies, and the consensus committee makes no assertions regards CTE in the presence of AD or other neurodegenerative disorders at this time."([10] page 218)* If one considers that the entirety of the cerebral cortex in advanced Alzheimer's disease (e.g., Braak stage VI [18]) is extensively involved by p-tau aggregates within neurons at essentially all sulcal depths and around all small blood vessels, it is not hard to imagine that identifying, and attaching significance to, a localised p-tau pattern resembling CTE-NC might be challenging in this setting. The authors of the 2021 consensus article identified this as an area of uncertainty with members of the Consensus panel being unable to reliably identify the 'stage' of CTE-NC using the "McKee Grading System". The group, for example, did not uniformly agree on CTE stage in any of 19 cases. Three different stages were assigned to 11 CTE cases, and in one case, pathologists offered all 4 CTE stages. Bieniek et al

write “Amongst the 8 raters, Kappa values were 0.22 and 0.19 for the blinded first round and unblinded second round, respectively” [10]. Interpretation of these values can vary but the well referenced Landis and Koch [19] would suggest slight (0.00-0.20) to fair (0.21-0.40) agreement. This is in addition to the validity concerns noted above, and the lack of specificity for associations of clinical symptomatology with CTE-NC in either the 2016 or 2021 definition. All of this serves to underscore the need for more research and the premature nature of causal assertions.

Lewy bodies (intraneuronal aggregates of alpha-synuclein) are sometimes invoked as an analogy for justifying a single microscopic lesion threshold for CTE-NC [20]. According to that thought process, the presence of a Lewy body indicates Lewy body disease, even if subclinical, just like CTE-NC equates to an encephalopathy, even if subclinical. A distinction here is that the end-stage clinical syndromes (e.g., Parkinson’s disease, Lewy body dementia) are well-defined neurological conditions, which is presently lacking with CTE-NC. Moreover, Lewy body “lesion burden” that might be validated against clinical progression is the Lewy body itself. The CTE burden (“high CTE” versus “Low CTE”) as proposed in the 2021 consensus article[10] “working protocol” is not based on the so-called pathognomonic CTE lesion, but rather the neurofibrillary tangle, a change otherwise indistinguishable from that observed in aging and Alzheimer’s disease. Clinical validation of such a protocol would have to consider potential confounding influences of simple aging and Alzheimer’s disease.

Finally, there is a general impression in the neuropathology community, reflected among the medical literature and media sources, that CTE-NC is restricted to contact sport athletes. We do not dispute that most cases of CTE-NC have been found in the sports population. Readers should keep in mind, however, that CTE-NC is not restricted to contact sport athletes or individuals with a TBI history. For example, Bieniek et al reported a study of 750 consecutive autopsy cases (the largest CTE-NC study to date) and found CTE-NC in 27 out of 300 former athletes (baseball, basketball, boxing, football, hockey soccer, wrestling, among other sports) versus 15 out of 450 *non-athletes* [21]. Two of the authors of that study also co-authored the 2021 CTE-NC consensus articles.

As another example, Noy et al, reported CTE-NC, (which they termed either “CTE-like” or “CTE”) in 39 out of 111 prospectively examined autopsies among individuals less than 60 years of age [21]. The relatively young cohort was selected to “avoid the complication of interpreting p-tau aggregates in the context of subclinical Alzheimer disease-type changes” [22]. Of the 111 individuals, 34 had microscopic lesions consistent with CTE-NC (as the pathology was described at the time the paper was written, which the authors termed “Stage <1 CTE” cases) and a further five cases were identified with “Stage 1 or Stage 2” CTE. Twenty eight of the 39 cases (72%) had histories of substance abuse, 59% had histories of brain trauma and 46% had histories of both substance abuse and brain trauma. Sixteen of the 39 cases (41%) with “CTE-like” or “CTE” pathology *had no history of head trauma*, of which 5 (13%) had histories of neither substance abuse nor head trauma. Ten of the 39 (26%) cases with no head trauma had histories of substance abuse [22].

Additional files 3. Misrepresentations of historical research

The paper also contains other specific misrepresentations of historical research. For example: "*CTE was described in boxers as early as the 1920s and by the 1950s it was widely accepted that hits to the head caused some boxers to become 'punch drunk.'"*[23]

> *"Dr. Harrison Martland is credited with first identifying the syndrome that was later called CTE in his article Punch Drunk, published in the Journal of the American Medical Association in 1928. Martland found evidence of traumatic brain injuries in the post-mortem brains of boxers and proposed a clinical syndrome based on his experience treating retired boxers (original reference is to Martland* [24]. *Pathologically, Martland described multiple 'ring hemorrhages' with perivascular distribution in the deeper structures of the brain."*[23]

The only post-mortem analysis described in Martland's 1928 article was that of a 76 year old male who stumbled and hit his head while going upstairs [24]. The man 'died thirty hours after admission to the hospital.' Martland illustrated this patient as a surrogate for his theory that concussion-related hemorrhages might be the cause of punch-drunk syndrome. With the benefit of subsequent knowledge of traumatic brain injury, the depicted gross neuropathology - *hemorrhage in the corpus callosum, gliding contusions, and small hemorrhages in the basal ganglia* - might be diagnosed as diffuse axonal injury and severe traumatic brain injury today. But as a factual matter, Martland's article did not describe 'evidence of traumatic brain injuries in the post-mortem brains of boxers'. The first post mortem examinations on boxers did not appear until the 1950's and even then were heterogeneous, and encompassed misdiagnosed neurodegenerative diseases in some cases (e.g., Alzheimer's disease, progressive supranuclear palsy, Lewy body dementia) [25].

The totality of the historical description presents a false narrative – that a forensic pathologist drew on his experience 'treating retired boxers' (for which no record exists), described post-mortem findings in boxers with progressive neurological decline (which he did not), and codified chronic traumatic encephalopathy as a uniform pathological process 'nearly a century' ago (which he did not). Readers are given the impression that modern day CTE-NC, drawing on historical and recent knowledge, is simply a refinement of an overarching condition. This is incorrect and misleading.

doi 10.1007/s40279-024-02102-4

Additional files 4. Case control studies and odds ratios

Additional files Table 2 presents an overview of the data required to calculate an Odds Ratio, with cells labelled A, B, C and D being of specific interest. These data are used to present two key pieces of information:

1. The odds that a CTE-NC case was exposed to RHI. (cell A divided by cell C)

2. The odds that a case without CTE-NC was exposed to RHI (cell B divided by cell D)

The division of these two pieces of information is the 'odds ratio' (the odds that a CTE-NC case was exposed to RHI *divided by* the odds that a case without CTE-NC was exposed to RHI).

In practice, a simplified equation is used to calculate the odds ratio: $\frac{A \times D}{B \times C}$.

In **Additional files Table 3**, the odds ratios presented in the review [23] have been re-created, demonstrating the data input that we believe are erroneous.

Additional files Table 2. Odds ratio calculation example for repetitive head impacts and chronic traumatic encephalopathy neuropathological change.

		**OUTCOME (Chronic traumatic encephalopathy neuropathological change)**		
		**CTE-NC +**	**CTE-NC -**	
**EXPOSURE (Repetitive head impacts)**	**Exposed**	Cell **[A]** Cohort with CTE-NC exposed to RHI.	Cell **[B]** Cohort without CTE-NC exposed to RHI	Total exposed to RHI
	**Not exposed**	Cell **[C]** Cohort with CTE-NC not exposed to RHI	Cell **[D]** Cohort without CTE-NC not exposed to RHI	Total not exposed to RHI
		Total with CTE-NC	Total without CTE-NC	Total of cohort

Additional files Table 3. Our understanding of the data used by in the review [23] to calculate odds ratios as evidence for the Strength of Association.

**(citation)**<br>**Article title**<br>**Journal of publication**	**Re-creation of published Odds Ratios**
[26]<br>The spectrum of disease in chronic traumatic encephalopathy.<br>Brain.	"A total of 85 brains from former athletes, military veterans or civilians with a history of repetitive mild traumatic brain injury" page 45<br>From the text:<br>A= positive cases, with exposure = 68<br>B= negative cases, with exposure =17<br>C= positive cases, without exposure = 0.5*<br>D= negative cases, without exposure = 18^<br><br>*There were no positive cases without exposure in the original study because all cases were selected based on their exposure to RHI.<br>^18 cases were cognitively intact individuals without history of mild TBI.
[27]<br>Duration of American football play and chronic traumatic encephalopathy.<br>Ann Neurol.	In the original study, only cases that were exposed to American football were included (not a case-control design). All cases were sourced from the VA-BU-CLF or FHS Brain Banks, limited to males who played American football and whose age at death was ≥20 years.<br>Total = 266<br>A= positive cases, with exposure = 223<br>B= negative cases, with exposure = 43<br>C= positive cases, without exposure = 0<br>D= negative cases, without exposure = 0<br>The odds ratio cannot be calculated without valid cases in the non-exposed group.
[28]<br>Chronic traumatic encephalopathy in the brains of military personnel.<br>N Engl J Med	All cases were chosen based on military service sourced from a military brain bank (not a case control study).<br>Total = 225<br>A= positive cases, with exposure = 10*<br>B= negative cases, with exposure = 50<br>C= positive cases, without exposure = 0.5^<br>D= negative cases, without exposure = 165<br><br>*among the 10 cases, half were "characterized by minimal neuropathologic changes"<br>^no positive cases without exposure
[21]<br>Association between contact sports participation and chronic traumatic encephalopathy: a retrospective cohort study.<br>Brain Pathol.	From 2566 autopsy cases, 300 former athletes and 450 non athletes were selected.<br>Total 750<br>A= positive cases, with exposure = 27*<br>B= negative cases, with exposure 300-27 = 273<br>C= positive cases, without exposure = 15*<br>D= negative cases, without exposure = 450 – 15 = 435<br><br>CTE= consistent with CTE consensus or features of CTE.

	*Of the 42 combined CTE pathology brains, 21 were classified as CTE-positive, consistent with established consensus criteria, while 21 displayed features of CTE.
[29]<br>Lewy body pathology and chronic traumatic encephalopathy associated with contact sports.<br>J Neuropathol Exp Neurol.	Three datasets in the original study:<br>1. The first dataset (UNITE) selected cases based on exposure.<br>2. The second dataset (FHS) has 1 CTE case and 19 cases with exposure to contact sports (from total of 164 included).<br>3. The third dataset appears was not in the analysis. There was no exposure data.<br>Values appear to be obtained from two of the three studies (UNITE and FHS) to calculate the OR.<br><br>269 (UNITE) were participants with history of exposure, 217 CTE<br>A= positive cases, with exposure = 217<br>B= negative cases, with exposure = 52<br>C= positive cases, without exposure = 0<br>D= negative cases, without exposure = 0<br><br>164 participants from the brain bank of the FHS, 1 with CTE<br>A= positive cases, with exposure= 0<br>B= negative cases, with exposure = 19 (from Table 1)<br>C= positive cases, without exposure = 1 (assumed as not exposed)<br>D= negative cases, without exposure = 144<br><br>261 participants from BU Alzheimer Disease Center (ADC) brain bank. No exposure case numbers presented, 7 CTE.
[30]<br>Chronic traumatic encephalopathy pathology in a neurodegenerative disorders brain bank.<br>Acta Neuropathol.	Records from 1,721 men identified 66 patients with history of exposure to contact sports. A control cohort of 198 disease-matched individuals (132 men and 66 women) was selected based upon age at death. None of these individuals had documented involvement in contact sports. Exposure should be *unknown* at selection to calculate the odds of a case in the exposed and non-exposed groups. These participants were selected based on their exposure (Neuropathologic controls included men without documented exposure to contact sports selected from the 1721 men identified in the screen of medical records.)<br><br>A= positive cases, with exposure = 21<br>B= negative cases, with exposure 45<br>(or if B includes 33 from the 'control' group, B then equals **78)**<br>C= positive cases, without exposure = ?? unknown because they were selected due to their exposure to contact sport<br>D= negative cases, without exposure (198 minus 33 who had 'exposure to head trauma' =165)<br>A = 21<br>B =78<br>C = 0.5*<br>D = 165<br>* zero positive cases without exposure

doi 10.1007/s40279-024-02102-4

REFERENCES

1 McIntosh AS, Patton DA, Fréchède B, *et al.* The biomechanics of concussion in unhelmeted football players in Australia: a case–control study. *BMJ Open*. 2014;4:e005078. doi: 10.1136/bmjopen-2014-005078

2 Cripton PA, Dressler DM, Stuart CA, *et al.* Bicycle helmets are highly effective at preventing head injury during head impact: Head-form accelerations and injury criteria for helmeted and unhelmeted impacts. *Accid Anal Prev*. 2014;70:1–7. doi: 10.1016/j.aap.2014.02.016

3 McIntosh AS. Evaluation of cricket helmet performance and comparison with baseball and ice hockey helmets. *Br J Sports Med*. 2003;37:325–30. doi: 10.1136/bjsm.37.4.325

4 McIntosh AS, Lai A, Schilter E. Bicycle Helmets: Head Impact Dynamics in Helmeted and Unhelmeted Oblique Impact Tests. *Traffic Inj Prev*. 2013;14:501–8. doi: 10.1080/15389588.2012.727217

5 McIntosh AS, Patton DA. Boxing headguard performance in punch machine tests. *Br J Sports Med*. 2015;49:1108–12. doi: 10.1136/bjsports-2015-095094

6 Sandmo SB, McIntosh AS, Andersen TE, *et al.* Evaluation of an In-Ear Sensor for Quantifying Head Impacts in Youth Soccer. *Am J Sports Med*. 2019;47:974–81. doi: 10.1177/0363546519826953

7 Mertz HJ, Irwin AL, Prasad P. Biomechanical and Scaling Basis for Frontal and Side Impact Injury Assessment Reference Values. 2016:2016-22–0018.

8 CEN/TR 16148 - Head and neck impact, burn and noise injury criteria - A Guide for CEN helmet standards committees | GlobalSpec. https://standards.globalspec.com/std/1379780/CEN/TR%2016148 (accessed 21 January 2024)

9 McKee AC, Cairns NJ, Dickson DW, *et al.* The first NINDS/NIBIB consensus meeting to define neuropathological criteria for the diagnosis of chronic traumatic encephalopathy. *Acta Neuropathol (Berl)*. 2016;131:75–86. doi: 10.1007/s00401-015-1515-z

10 Bieniek KF, Cairns NJ, Crary JF, *et al.* The Second NINDS/NIBIB Consensus Meeting to Define Neuropathological Criteria for the Diagnosis of Chronic Traumatic Encephalopathy. *J Neuropathol Exp Neurol*. 2021;80:210–9. doi: 10.1093/jnen/nlab001

11 Braak H, Del Tredici K. The pathological process underlying Alzheimer's disease in individuals under thirty. *Acta Neuropathol (Berl)*. 2011;121:171–81. doi: 10.1007/s00401-010-0789-4

12 Brett BL, Wilmoth K, Cummings P, *et al.* The neuropathological and clinical diagnostic criteria of chronic traumatic encephalopathy: a critical examination in relation to other neurodegenerative diseases. *J Alzheimers Dis*. 2019;68:591–608. doi: 10.3233/JAD-181058

13 P. Gelber R, J. Launer L, R. White L. The Honolulu-Asia Aging Study: Epidemiologic and Neuropathologic Research on Cognitive Impairment. *Curr Alzheimer Res*. 2012;9:664–72. doi: 10.2174/156720512801322618

14 McKee AC, Mez J, Abdolmohammadi B, *et al.* Neuropathologic and Clinical Findings in Young Contact Sport Athletes Exposed to Repetitive Head Impacts. *JAMA Neurol*. 2023;80:1037. doi: 10.1001/jamaneurol.2023.2907

15 Mez J, Alosco ML, Daneshvar DH, *et al.* Validity of the 2014 traumatic encephalopathy syndrome criteria for CTE pathology. *Alzheimers Dement*. 2021;17:1709–24. doi: 10.1002/alz.12338

16 Iverson GL, Kissinger-Knox A, Huebschmann NA, *et al.* A narrative review of psychiatric features of traumatic encephalopathy syndrome as conceptualized in the 20th century. *Front Neurol*. 2023;14:1214814. doi: 10.3389/fneur.2023.1214814

17 Iverson GL, Castellani RJ, Cassidy JD, *et al.* Examining later-in-life health risks associated with sport-related concussion and repetitive head impacts: a systematic review of case-control and cohort studies. *Br J Sports Med*. 2023;57:810–21. doi: 10.1136/bjsports-2023-106890

18 Braak H, Alafuzoff I, Arzberger T, *et al.* Staging of Alzheimer disease-associated neurofibrillary pathology using paraffin sections and immunocytochemistry. *Acta Neuropathol (Berl)*. 2006;112:389–404. doi: 10.1007/s00401-006-0127-z

19 Landis JR, Koch GG. The measurement of observer agreement for categorical data. *Biometrics*. 1977;33:159–74.

20 McKee AC. Author's Reply: *J Neuropathol Exp Neurol*. 2014;73:375. doi: 10.1093/jnen/73.4.375-a

21 Bieniek KF, Blessing MM, Heckman MG, *et al.* Association between contact sports participation and chronic traumatic encephalopathy: a retrospective cohort study. *Brain Pathol*. 2020;30:63–74. doi: 10.1111/bpa.12757

22 Noy S, Krawitz S, Del Bigio MR. Chronic Traumatic Encephalopathy-Like Abnormalities in a Routine Neuropathology Service. *J Neuropathol Exp Neurol*. 2016;75:1145–54. doi: 10.1093/jnen/nlw092

23 Nowinski CJ, Bureau SC, Buckland ME, *et al.* Applying the Bradford Hill Criteria for Causation to Repetitive Head Impacts and Chronic Traumatic Encephalopathy. *Front Neurol*. 2022;13:938163. doi: 10.3389/fneur.2022.938163

24 Martland HS. Punch Drunk. *J Am Med Assoc*. 1928;91:1103–7. doi: 10.1001/jama.1928.02700150029009

25 Goldfinger MH, Ling H, Tilley BS, *et al.* The aftermath of boxing revisited: identifying chronic traumatic encephalopathy pathology in the original Corsellis boxer series. *Acta Neuropathol (Berl)*. 2018;136:973–4. doi: 10.1007/s00401-018-1926-8

26 McKee AC, Stein TD, Nowinski CJ, *et al.* The spectrum of disease in chronic traumatic encephalopathy. *Brain*. 2013;136:43–64. doi: 10.1093/brain/aws307

27 Mez J, Daneshvar DH, Abdolmohammadi B, *et al.* Duration of American Football Play and Chronic Traumatic Encephalopathy. *Ann Neurol*. 2020;87:116–31. doi: 10.1002/ana.25611

28 Priemer DS, Iacono D, Rhodes CH, *et al.* Chronic Traumatic Encephalopathy in the Brains of Military Personnel. *N Engl J Med*. 2022;386:2169–77. doi: 10.1056/NEJMoa2203199

29 Adams JW, Alvarez VE, Mez J, *et al.* Lewy body pathology and chronic traumatic encephalopathy associated with contact sports. *J Neuropathol Exp Neurol*. 2018;77:757–68. doi: 10.1093/jnen/nly065

30 Bieniek KF, Ross OA, Cormier KA, *et al.* Chronic traumatic encephalopathy pathology in a neurodegenerative disorders brain bank. *Acta Neuropathol (Berl)*. 2015;130:877–89. doi: 10.1007/s00401-015-1502-4
